# Parasite management in aquaculture exerts selection on salmon louse behaviour

**DOI:** 10.1111/eva.13255

**Published:** 2021-06-02

**Authors:** Andrew Coates, Ingrid A. Johnsen, Tim Dempster, Ben L. Phillips

**Affiliations:** ^1^ Sustainable Aquaculture Laboratory – Temperate and Tropical (SALTT) School of BioSciences University of Melbourne Parkville Vic. Australia; ^2^ Spatial Ecology and Evolution Lab (SPEEL) School of BioSciences University of Melbourne Parkville Vic. Australia; ^3^ Institute of Marine Research Bergen Norway

**Keywords:** aquaculture, copepodid, dispersal model, louse, salmon, selection

## Abstract

The evolution of pest resistance to management strategies is a major challenge for farmed systems. Mitigating the effects of pest adaptation requires identifying the selective pressures imposed by these strategies. In Atlantic salmon (*Salmo salar*) aquaculture, barriers are used to prevent salmon louse (*Lepeophtheirus salmonis*) larvae (copepodids) from entering salmon cages. These barriers are effective against shallow‐swimming copepodids, but those swimming deeper can pass underneath and infest salmon. Laboratory experiments suggest that depth regulation in copepodids is a variable behavioural trait with a genetic basis. We used biological–hydrodynamic dispersal models to assess how this trait variation alters the dispersion of lice through the ocean environment and into farms. The dispersal of copepodids with 3 behavioural phenotypes (*deep,* *mean* or *shallow*) was modelled over winter–spring and spring–summer periods in a Norwegian fjord system with intensive aquaculture. The infestation pressure of each phenotype on barrier cages was estimated from their modelled depth distributions: copepodids deeper than 10 m were predicted to successfully pass underneath barriers. The *deep* phenotype was the most abundant below 10 m and reached infestation pressures 3 times higher than that of the *mean* phenotype. In contrast, the *shallow* phenotype infestation pressure reached less than half that of the *mean* phenotype. These differences in relative fitness indicate that barriers can impose strong directional selection on the swimming behaviour of copepodids. The strength of this selection varied seasonally and geographically, with selection for the *deep* phenotype stronger in winter–spring and at coastal locations than in spring–summer and within fjords. These findings can be applied across farms to slow louse adaptation, by limiting barriers during situations of strong selection, although this must be balanced against trade‐offs to short‐term efficacy. More broadly, our study highlights new ways in which dispersal models can address evolutionary questions crucial for sustainable parasite management in aquaculture.

## 1 INTRODUCTION

Farming is inextricably tied to evolutionary processes, and this is particularly true for pest management (Thrall et al., 2011). Pest populations are not static, but often adapt to control measures, such as pesticides, biological control and crop rotation (Georghiou & Saito, 1983; Krysan et al., 1986; Levine et al., 2002; Tomasetto et al., 2017; Whalon et al., 2008). The long‐term efficacy of pest management strategies can be safeguarded against resistance if they are coordinated according to evolutionary principles (Barzman et al., 2015; Stear et al., 2001; Zhan et al., 2015). For example, combining multiple treatments and maintaining treatment‐free refuges can slow pest adaptation, by reducing the overall strength of selection imposed on the pest population (Carrière et al., 2010; REX Consortium, 2013; Rimbaud et al., 2018). Identifying selection pressures imposed by a management strategy is therefore a key step towards ensuring the durability of that strategy. Are certain traits selected for? How strong is this selection? Is selection heterogeneous across space or time?

These questions are particularly salient for salmon aquaculture. The ectoparasitic copepod *Lepeophtheirus salmonis* (salmon louse) presents a major challenge to the sustainable growth of the salmon aquaculture industry (Torrissen et al., 2013). The productivity and welfare of farmed salmon are adversely affected by louse infestations, as well by the methods used to treat infestations (Finstad et al., 2000; Grimnes & Jakobsen, 1996; Overton et al., 2019; Wagner et al., 2008). Outbreaks of lice on farms also impact wild salmonid populations (Bjørn et al., 2001; Krkošek & Hilborn, 2011; Krkošek et al., 2013). Given the size and value of the salmon aquaculture industry (FAO, 2018), there has been considerable incentive for the development of effective louse controls. The efficacy of chemical pesticides has declined significantly in many areas, due to rapid adaptation by salmon lice (Aaen et al., 2015). As a consequence, farms have reduced chemical use and shifted to various nonchemical strategies, including ‘depth‐based preventions’ (Barrett et al., 2020; Coates et al., 2021). Unlike other major nonchemical strategies, which treat salmon already infested with lice, depth‐based preventions reduce the chances of copepodids (the infective, transmissive larval stage of lice) infesting a salmon cage in the first place. Copepodids typically aggregate at shallow depths in the water column, which is where infestations are most likely to occur (Penston et al., 2008; Salama et al., 2018; Samsing, Johnsen, et al., 2016).

The most widely used approaches to depth‐based prevention (and those with the strongest evidence of efficacy) are cage barriers technologies (Barrett et al., 2020). Barriers encircling the upper levels of a sea‐cage impede larval access from surface waters, whilst leaving deeper parts of the cage open to allow sufficient water circulation (Grøntvedt et al., 2018; Stien et al., 2018; Wright et al., 2017). Although depth‐based preventions can reduce infestations by 75% or more, some copepodids manage to pass underneath these barriers and infest a cage (Geitung et al., 2019; Oppedal et al., 2017; Stien et al., 2018).

The depth of infective copepodids is a product of environmental forces (such as currents and hydrodynamic mixing; Johnsen et al., 2014) and their vertical swimming behaviour in response to cues such as pressure, salinity and light (Coates et al., 2020; Crosbie et al., 2019; Heuch et al., 1995). Individual variation in swimming behaviour—and hence depth—is regularly observed in small‐scale experimental columns (Bricknell et al., 2006; Crosbie et al., 2019; Heuch, 1995). Coates et al. (2020) found a significant effect of family on this variation: lice from some families were more likely to aggregate at the bottom of columns than others. Differences were consistent when columns were pressurized to simulate the conditions found at a range of depths down to 10 m. Overall, higher pressures stimulated an increased frequency of upwards swimming, but the strength of this response varied strongly between families. There was no clear evidence of environmental or maternal effects accounting for this variation, pointing to at least some heritable component to vertical swimming behaviour in copepodids, although more research is needed to quantify this (Coates et al., 2020).

Assuming a genetic element to this behavioural variation, if depth‐based preventions select for louse copepodids that occur deeper in the water column (those that pass underneath barriers), adaptive responses are likely to ensue. To assess this possibility, it is first essential to determine whether the inter‐family variation observed in experimental columns (Coates et al., 2020) translates to differences in depth in the natural environment. Differences in the distribution of families might be diluted by the much larger scale of the natural water column or by environmental factors such as currents and salinity. Experiments using large scale, *in situ* columns (e.g. as used in Tang et al., 2011) is one approach for testing this, but performing sufficient replicates with this method across a realistic range of natural conditions would be prohibitively costly and time‐consuming. An alternative approach is to use computer simulations to model louse distributions over large spatial and temporal scales.

Sea lice dispersal models predict the dispersion of larvae through the environment and are powerful tools for researching and monitoring louse transmission dynamics (Asplin et al., 2014; Johnsen et al., 2016; Murray & Gillibrand, 2006). By predicting the infestation pressure of farm outbreaks on wild salmonid populations, these models inform decisions on how salmon aquaculture is managed across Norway (Myksvoll et al., 2018, 2020; Vollset et al., 2018). Here, we use a louse dispersal model for a novel, evolutionary purpose: to predict the strength of selection imposed on lice by the widespread use depth‐based preventions. Practical applications of this model include informing decisions on how preventative strategies can be coordinated across farms to protect against resistance.

Our first aim was to assess whether inter‐family variation in swimming behaviour influences the vertical and horizontal dispersion of copepodids in the natural environment. To do so, we converted behavioural data (from Coates et al., 2020) into new parameters for a Norwegian lice dispersal model and tracked the effect of different behaviours on the spatial distribution of larvae in three dimensions. We then used these outputs to estimate the strength of selection that depth‐based preventions might impose on louse behavioural phenotypes. Finally, we examine how the strength of this selection varies over time and space.

## 2 METHODS

### 2.1 Salmon louse biology

There are eight stages in the *Lepeophtheirus salmonis* life cycle (Hamre et al., 2019). The first three (two nauplius stages and one copepodid stage, referred collectively here as ‘larvae’) are planktonic and can be transported on currents for tens of kilometres (Asplin et al., 2014; Samsing, Johnsen, et al., 2016). Nauplii are noninfective, whereas copepodids will seek out and attach to a host, whereupon they will complete the life cycle as a parasite. Since larvae are nonfeeding, copepodids have a limited time window to find a host before their yolk reserves are depleted (Samsing, Oppedal, et al., 2016). It is through the dispersive copepodid phase that salmon lice are transmitted to farms from the external environment.

### 2.2 Methods overview

Experimental data on copepodid distributions (from Coates et al., 2020) were converted into parameters for the swimming behaviour of different copepodid phenotypes. These parameters were then incorporated into a lice dispersal model, to simulate the vertical and horizontal distribution of each phenotype through the study area. The modelled abundance and depth of copepodids were used to predict the efficacy of depth‐based preventions, for each phenotype at various spatial and temporal scales. We estimated the relative fitness of each phenotype based on their success at infesting farms using depth‐based preventions. Finally, selection gradients were calculated (from a regression of phenotype against relative fitness) to predict the strength of the selection imposed by prevention strategies.

### 2.3 Constructing behavioural parameters from experimental data

Based upon the findings of Coates et al. (2020), we parameterized vertical swimming behaviour to reflect (1) that copepodids regularly alternate between ‘swim’ and ‘sink’ states and (2) that the probability of a copepodid being in a state is a function of its depth and its genetic background. Using data from Coates et al. (2020), we calculated the proportion of copepodids in ‘swim’ and ‘sink’ states, for each family tested and for each simulated depth (0, 5 and 10 m). We designated copepodids in the top and bottom of columns into ‘swim’ and ‘sink’ states, respectively; of the remainder in the middle of columns, we assigned half to each state. We assumed that the proportion of individuals in one state at a given point in time is equal to the probability of any one individual being in that state. In our dispersal model, lice were assigned a probability of moving up (*p_z_*) or down (1−*p_z_*) at each timestep, which was dependent on the particles' depth (*z*) and family background (Coates et al., 2020). We modelled 3 distinct behavioural phenotypes, reflecting variation between families: *mean*, *deep* and *shallow* phenotypes. For the *mean* phenotype, we calculated the mean *p_z_* across all (*n* = 37) families tested in Coates et al. (2020). For the *deep* phenotype, we took the 4 families with the lowest *p_z_* values (consistent across depths) and calculated the mean of these values. We did the same for the 4 families with the highest *p_z_* values to give us the *shallow* phenotype (Table 1). In this way, we captured the mean swimming behaviour, and the behaviours of the ~10% deepest‐distributed and ~10% shallowest‐distributed families. We then constructed separate probability curves that best fit the *p_z_* values for each family, using a Michaelis–Menten type equation:

(1) $$p_{z}=\frac{{\left(\left(p_{10}+0.01\right)‐p_{0}\right)}^{z}}{K_{M}+z}+p_{0}$$

**TABLE 1:** The probability of copepodids swimming upwards (*p_z_*), for 3 behavioural phenotypes and at 3 depths (*z*; 0, 5 and 10 m)

Behavioural phenotype	Proportion of copepodids swimming (*p_z_*) at metres depth (*z*)			*K_M_*
	*p_0_*	*p_5_*	*p_10_*	
Deep	0.33	0.44	0.43	1
Mean	0.46	0.61	0.61	0.5
Shallow	0.65	0.78	0.80	1

Note

*K_M_* is the depth (nearest 0.5 m) at which $p_{z}=\frac{p_{10}}{2}$.

Values of *p_0_* and *p_10_* (the probability of swimming at 0 and 10 m depth); and *K_M_* (the value of *z* at which $p_{z}=\frac{p_{10}}{2}$) are given in Table 1 (see Figure S1 for curve fits).

Louse larvae avoid low‐salinity water close to the surface by actively swimming down to more saline depths (Crosbie et al., 2019). We included this behaviour in our model, based on Sandvik et al. (2020). When lice resided in water with a salinity <31 ppt, Equation 1 was overridden by a new function:

(2) $$p_{d}=\frac{31}{8}‐\frac{s}{8}$$

where the probability of swimming downwards (*p_d_*) is a function of salinity (*s*, in ppt). As *s* decreases from 31 to 23 ppt, the likelihood of downwards swimming increases linearly to 1. When salinity is <23 ppt, lice will always swim down.

At every timestep (*δt* = 120 s) in the dispersal model, a value of *p_z_* (or *p_d_*) was calculated for each individual louse particle according to its family, depth and surrounding salinity. Realized swimming state was drawn from a Bernoulli distribution given by *p_z_*. For example, if *p_z_* = 0.75, a particle would have a 75% chance of moving up and a 25% chance of moving down.

In keeping with the standard simulation, all simulations also included random vertical movement to simulate fine‐scale hydrodynamic mixing. This was given by the random walk:

(3) $$\delta w_{R}=R\sqrt{\frac{2D}{\delta t}}$$

where *δw_R_* is the random velocity, *D* is the diffusion coefficient (10^−3^ m^2^ s^−1^), and the random number *R* is drawn for every particle each timestep from a normal distribution with a mean of 0 (Johnsen et al., 2014).

### 2.4 Lice dispersal model

These behavioural parameters were incorporated into a salmon lice dispersal model. The full parameterization of this model is described in detail elsewhere (Asplin et al., 2014; Johnsen et al., 2016; Myksvoll et al., 2018). In brief, the model system combines two components: an ocean circulation model and an individual‐based particle‐tracking model. The ocean circulation model simulates hydrodynamic processes in a study area. We ran simulations for the Hardangerfjord area, on the south‐west coast of Norway (60° N, 5.5° E). This is a region of high‐intensity aquaculture, with almost 200 approved farm sites selling >200,000 tonnes of salmonids in 2019 (http://fiskeridir.no; http://barentswatch.no). It consists of a complex system of branching fjords (extending ~180 km inland), islands and coastline; with spatially and temporally heterogeneous hydrodynamics (Asplin et al., 2014; Johnsen et al., 2016). We obtained information on the currents, temperature and salinity of this area using a downscaled version of the NorKyst800 ocean circulation model, which is an implementation of the Regional Ocean Model System (Albretsen et al., 2011; Shchepetkin & McWilliams, 2005). Simulations were run with a horizontal resolution of 160 m × 160 m and with 35 vertical layers. The ocean model predictions for Hardangerfjord closely match the field data, with temperature, salinity and current flow values typically deviating from observations by 1°C, 1 ppt and 0.02 ms^−1^ at most (Asplin et al., 2020). In particular, the model predictions coincide well with observations during periods of strong forcing (for instance, strong wind episodes) and therefore recreate circular patterns important for estimating louse distribution and transport distances (Dalsøren et al., 2020).

The particle‐tracking model simulates the transport of particles (representing planktonic louse larvae) through the study area, as driven by the ocean circulation model. Particles were released from positions corresponding to farm locations. Their dispersal was simulated using the 4^th^ order Runge–Kutta scheme, solving for the Lagrangian equation of motion. The horizontal and vertical position of particles was calculated using a 120 s timestep and saved every hour. The water's surface acted as a reflecting border, whereas the shoreline was set as a nonreflecting border. The particles were given no depth limitation.

Particles were simulated to have the life history and behaviour of lice. Particles were released as ‘nauplii’ and underwent temperature‐dependent development, becoming ‘copepodids’ at 40 degree‐days (Samsing, Oppedal, et al., 2016). The lifespan and infectivity of copepodids were parameterized as a function of age and temperature in accordance with Skern‐Mauritzen et al. (2020). Background mortality was set at a constant rate of 17% day^−1^ (as estimated by Stien et al. 2005). Particles were given the ability to move up and down the water column (with a velocity of 0.5 mm s^−1^), according to our new behavioural parameters. The predicted infestation pressure from the particle‐tracking model in the Hardangerfjord area correlates strongly with infestation levels observed using sentinel cages (Sandvik et al., 2016, 2020) and on wild salmon (Johnsen et al., 2021; Myksvoll et al., 2018).

### 2.5 Simulation

**FIGURE 1:**
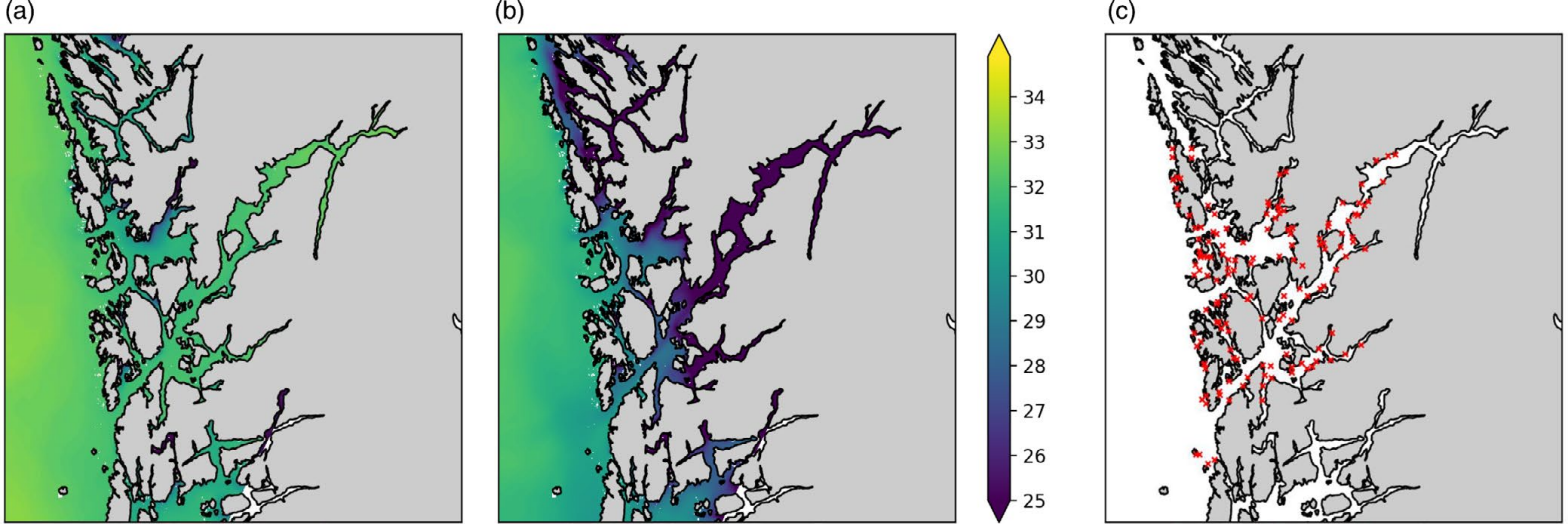
Surface salinity (ppt) in the Hardangerfjord area, Norway, during winter–spring March 2018; (a) and spring–summer May 2018; (b) simulations. Location of 144 farm sites (c)

To reflect seasonal effects on the dispersion of different louse families, we ran simulations for two periods: 1 January–31 March 2018 (‘winter–spring’) and 1 May–31^st^ July 2018 (‘spring–summer’). The average water temperature, as experienced by lice particles during the simulations, was 4.5°C in winter–spring and 11.6°C in spring–summer. In winter–spring, the surface salinity remained relatively high (32–33 ppt) through the study area (Figure 1a). In spring–summer, on the other hand, surface salinity was lower, particularly further into the fjord system (25 ppt; Figure 1b).

In each simulation, 5 particles (each representing 100 lice) were released every hour from each of 144 farm sites in the area (Figure 1c) to test inter‐family differences in dispersion. Particles were modelled with the behaviour of either the *mean*, *deep* or *shallow* phenotype, in separate simulations. A total of 6 simulations were run (3 phenotypes × 2 seasons).

The first month was used as a spin‐up period (i.e. particle data not considered in further calculations), to allow time for lice to develop into copepodids. We plotted the vertical distributions of copepodids with a 1 m resolution. The simulated horizontal distribution of lice was given by the average concentration of infestive copepodids per grid cell. The duration and infestation level of the copepodid stage was parameterized as a function of age and temperature, in accordance with (Skern‐Mauritzen et al., 2020). The hourly concentration of infective copepodids at farm sites were averaged over the 2‐month simulation period to estimate the average infestation pressure on farms (assuming no cage barriers).

### 2.6 Estimating barrier efficacy and relative fitness of phenotypes

To estimate the infestation pressure on farms, assuming they were using 10‐m‐deep cage barriers, we multiplied the farm infestation pressure by the proportion of copepodids deeper than 10 m. These values were assumed to provide a good metric of fitness, as the survival and reproduction of lice are dependent on the copepodid encountering a host, the vast majority of which are found on farms (Dempster et al., comparing farm stocks with estimates of wild population size). For each season and location, the barrier cage infestation pressure of each phenotype was divided by that for the *mean* phenotype, to calculate the relative fitness of each phenotype (relative to the *mean* phenotype). To assess for spatial variation in copepodid distribution and infestation pressure, we repeated the above for clusters of 10 farms in each of 3 different locations in the simulation area: inner‐fjord, mid‐fjord and coastal locations (see Figure S2).

### 2.7 Calculating the selection gradient to estimate selection strength

One approach to quantifying the strength of selection is to calculate the linear selection gradient (β), which is the slope of a linear regression of phenotype against relative fitness (Lande & Arnold, 1983). We used *p_10_* values (the probability of a copepodid being in the ‘swim’ state at 10 m depth, Table 1) for the 3 phenotypes as a metric of phenotype, and the relative (to *mean* phenotype) fitness as described above. Linear regressions were fit and the gradients (β) calculated using the statistics package *R* (Figure 5). Selection gradients are typically standardized by taking the absolute value and multiplying it by either the phenotypic trait mean (|βHereford et al., 2004; Matsumura et al., 2012) or the phenotypic standard deviation (|βKingsolver et al., 2001; Kingsolver & Pfennig, 2007). Across the 37 families tested in Coates et al. (2020), the family *p_10_* values were normally distributed (Shapiro–Wilk test of normality: *p* = 0.49) with mean of 0.61 and a standard deviation of 0.125. We used these values to calculate and compare |β_µ_| and |β_σ_|.

## 3 RESULTS

### 3.1 Vertical and horizontal distributions

**FIGURE 2:**
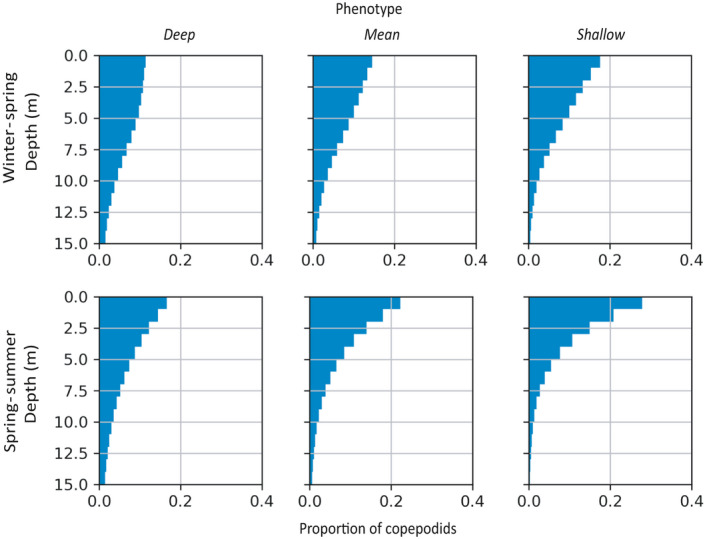
Vertical profiles of copepodids (proportion of individuals within 1 m increments) from *deep*, *mean* and *shallow* phenotypes; throughout winter–spring and spring–summer simulations

In all simulations, copepodids were most abundant close to the surface and diminished in number with increasing depth (Figure 2). Copepodids were distributed higher in the water column in winter–spring than in spring–summer: 73% of copepodids in winter–spring, and 61% in spring–summer (averaged across phenotypes) occurred in the top 5 m. Vertical distributions differed between behavioural phenotypes. Lice modelled with the *deep phenotype* were found at a greater abundance in deeper water than the other phenotypes (Figure 2). In winter–spring, for example, 75% of *deep* phenotype copepodids were found in the top 7 m of the water column, whereas 75% of the *shallow* phenotype occurred in just the top 4 m.

In both periods, the average horizontal transport distance was highest for the *shallow* phenotype (25.1 km in spring–summer, 37.8 km in winter–spring) and lowest for the *deep* phenotype (23.0 km and 36.8 km), although differences between phenotypes were small. These are the average distances from the release position during the entire copepodid lifespan.

### 3.2 Infestation pressure

**FIGURE 3:**
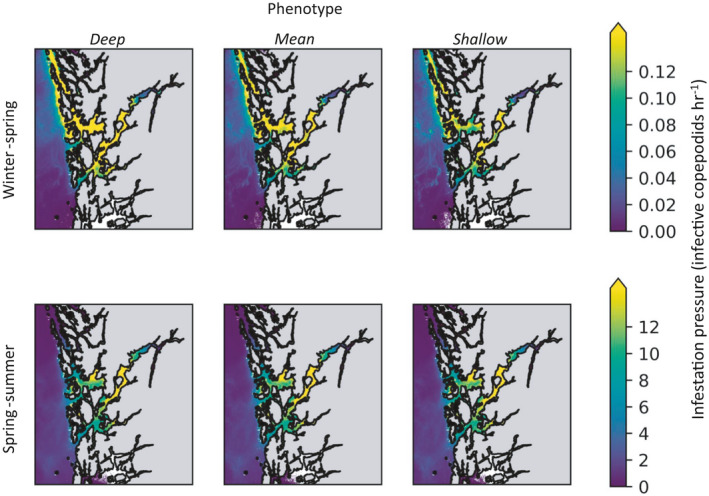
Infestation pressure (infective copepodids hr^−1^, aggregated over 2 months) across simulation area in winter–spring and spring–summer, for *deep*, *mean* and *shallow* phenotypes

In spring–summer, the infestation pressure (number of infective copepodids per grid cell per hour) at farms was similar for each phenotype. In winter–spring, the average infestation pressure across all farms was highest for the *deep* phenotype (22% higher than the *mean* phenotype) and lowest for the *shallow* phenotype (12% lower than the *mean* phenotype). Differences between phenotypes were consistent for inner‐fjord, mid‐fjord and coastal farm locations (Figure 3). Overall, copepodids tended to reach higher concentrations further into fjords, with the infestation pressure at inner‐fjord locations more than double that at coastal sites (Figure 3). The total infestation pressure in winter–spring was only ~1% of that in spring–summer.

### 3.3 Efficacy of depth‐based preventions

**FIGURE 4:**
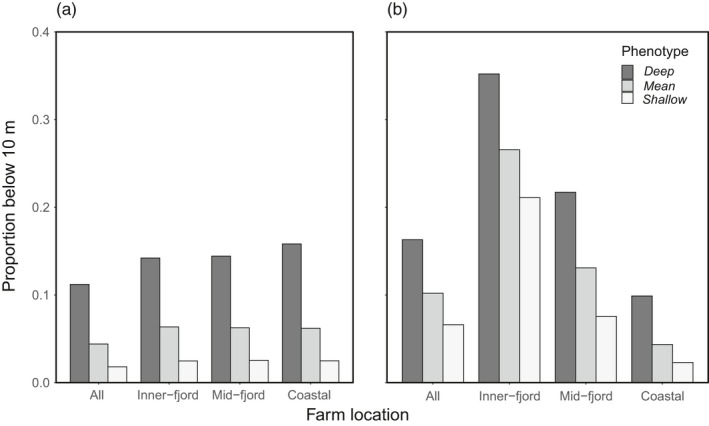
Proportion of copepodids at farm sites (at all farms, and at select inner‐fjord, mid‐fjord and coastal locations) deeper than 10 m depth, in winter–spring (a) and spring–summer (b) simulations

Of the infective copepodids occurring at farm sites, we calculated the percentage that were below 10 m depth. These percentages were highest for the *deep* phenotype and lowest for the *shallow* phenotype, at all places and times. In spring–summer, 16% of *deep* phenotype copepodids were deeper than 10 m, which was 1.6 and 2.5 times higher than for the *mean* and *shallow* phenotypes, respectively (Figure 4). In winter–spring, these percentages were lower, but the differences between phenotypes were even greater. In winter–spring, 11% of *deep* phenotype copepodids were below 10 m, which was 2.5 and 6.2 times higher than for the *mean* and *shallow* phenotypes, respectively (Figure 4).

In spring–summer, copepodids at inner‐fjord locations were more likely to occur below 10 m (e.g. 35% of the *deep* phenotype) than coastal locations (10% of the *deep* phenotype; Figure 4). The relative differences between phenotypes were greatest at coastal sites, however, with the percentage of *deep* phenotype lice more than twice that for the *mean* phenotype in spring–summer. In winter, the percentage of each phenotype below 10 m was similar across the three farming locations.

### 3.4 Relative fitness and selection gradients

**FIGURE 5:**
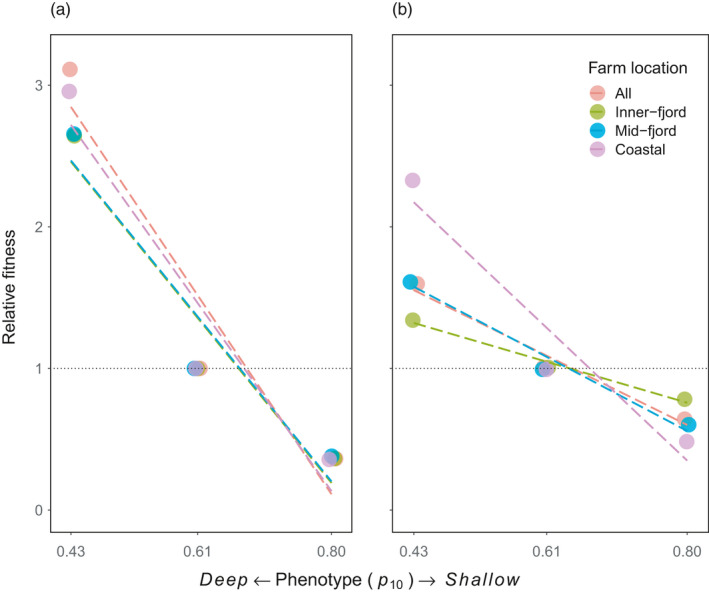
Relative fitness of copepodid phenotypes (relative to *mean* phenotype) in (a) winter–spring and (b) spring–summer, and at different farm locations. Phenotypes given as *p_10_* values (Table 1; with smaller values for a *deep* phenotype). Lines indicate linear regressions for data points

We multiplied the infestation pressure on farms by the proportion of lice deeper than 10 m, to predict the infestation pressure on farms using depth‐based preventions. For each season and farm location, we divided these values by those for the *mean* phenotype, to estimate the relative fitness of each phenotype (relative to the *mean* phenotype; Figure 5).

In winter–spring, the fitness of the *deep* phenotype was approximately 3 times that of the *mean* phenotype and 9 times that of the *shallow* phenotype. The standardized selection gradients indicate strong directional selection (|β_µ_| = 4.5; |β_σ_| = 0.95; Figure 5). The relative fitness of the *deep* phenotype was lower in spring–summer (1.6 times the *mean* phenotype), as were the selection gradients (|β_µ_| = 1.6, |β_σ_| = 0.33). The estimated selection gradient across the whole simulation area and both seasons was |β_µ_| = 3.0 and |β_σ_| = 0.64. In both seasons, the relative fitness of the *deep* phenotype was higher at coastal sites than at fjord locations, particularly in spring–summer (2.3 vs. 1.3; Figure 5).

## 4 DISCUSSION

Our simulations suggest that variation in the swimming behaviour of larval salmon lice, as observed in small‐scale experimental columns (Coates et al., 2020), translates to variation in the depth of lice in the natural environment. Copepodids modelled with the *deep* phenotype (representing the 10% of families with the deepest distributions in Coates et al., 2020) were vastly more abundant in deeper waters than the other phenotypes. Our model builds off a previously validated lice dispersal model (Sandvik et al., 2020), covers a physical and temporal scale that dwarfs that possible in the laboratory and included key environmental factors that influence the distribution of copepodid at different depths (e.g. currents, hydrodynamic mixing and salinity gradients). Our results indicate that even accounting for these environmental factors, variation in swimming behaviour produces large differences in the depth profile of copepodids. Our simulations suggest that the depth‐based prevention implemented on farms impose strong directional selection on louse behavioural phenotypes.

### 4.1 Efficacy of depth‐based preventions

Most depth‐based barriers used on commercial farms extend to ~10 m depth (e.g. Stien et al., 2018; Geitung et al., 2019). We assumed that copepodids above this depth were excluded by these barriers, whereas those below could pass underneath and infest caged fish. These assumptions are supported by Samsing, Johnsen, et al. (2016), who illustrated that lice dispersal models could be used to predict the efficacy of depth‐based preventions. In that study, the authors recorded louse infestations on salmon held at a range of depths in barrier cages and compared the data with predictions from a lice dispersal model (for the same place and time). In their simulations, 48% of lice occurred at depths ≥5 m, which closely matched with the observation that 40% of infestations occurred occurring in cages ≥5 m deep.

We predicted that widespread use of depth‐based preventions in the Hardangerfjord region could reduce louse infestation pressure by 73–96% (Figure 4, assuming the population is predominantly the *mean* phenotype). These are at the high end of the efficacies reported from barrier cage trials in similar locations and times of the year as our simulations (Geitung et al., 2019; Oppedal et al., 2017; Wright et al., 2017).

The % efficacy of depth‐based preventions was lower in spring–summer than in winter–spring (Figure 4). This was due to lower salinity at the surface, resulting from increased freshwater input during the spring melt (Figure 1; Asplin et al., 2020). Louse larvae move deeper in the water column to avoid brackish water (Bricknell et al., 2006; Crosbie et al., 2019); since this behaviour was included in our model, more copepodids (regardless of phenotype) sank below 10 m during times of low salinity. This was most pronounced in spring–summer at inner‐fjord sites, where a relatively thick, stable brackish layer accumulated (Figure 1). Experimental trials have shown that seasonal and spatial variation in salinity has a strong effect on the efficacy of depth‐based preventions (Geitung et al., 2019; Oppedal et al., 2019).

The predicted efficacy of depth‐based preventions differed for louse behavioural phenotypes, with these strategies being less effective against the *deep* phenotype. Although the absolute difference in efficacies is small (Figure 4), the relative differences between phenotypes are striking, with the estimated fitness of the *deep* phenotype reaching three times that of the *mean* phenotype, and eight times that of the *shallow* phenotype (Figures 4 and 5). Changes in relative fitness, rather than in absolute values, are the driver of evolutionary change, and our results indicate that depth‐based preventions impose strong directional selection on copepodid swimming behaviour. This is supported by the estimates of standardized selection gradients. Kingsolver et al. (2001) and Hereford et al. (2004) reviewed the literature on phenotypic selection and compiled estimates of |β| for various traits in natural systems. Our estimates (|β_µ_| = 3.0; |β_σ_| = 0.64, across both seasons) fall well above the median |β| values in these studies (|β_µ_| = 1.45; |β_σ_| = 0.16) and are indicative of very strong selection.

It must be noted that we used an unconventional approach to estimate these selection gradients. Fitness is usually scored on a continuous scale (e.g. the number of offspring produced), and the selection gradient is calculated using a data point for every individual in the population. In our system, individual fitness is binary: individuals either infest a cage (if they are deeper than 10 m at a farm site) or they do not infest a cage. We instead used data points representing the mean relative fitness of each phenotype, which should produce a slope representing the whole population (Figure 5).

### 4.2 Possible evolutionary response by lice to depth‐based preventions

Taken alongside preliminary evidence that swimming behaviour has a genetic component (Coates et al., 2020), our results support the hypothesis that directional selection by depth‐based preventions could shift the vertical distribution of the louse population. Similar shifts towards deeper distributions have evolved in other planktonic crustaceans in response to selection by predators (Cousyn et al., 2001; Gliwicz, 1986; De Meester, 1993, 1996). In lice, adaptation could lead to populations becoming resistant to barrier cages (Coates et al., 2021). Strong selection can drive rapid evolution, provided that the trait under selection is heritable (Falconer & Mackay, 1996).

The magnitude of our selection gradients |β| suggests that widespread use of barriers cages could drive a sizeable evolutionary response, even if genetic variation plays only a small role in the population's phenotypic variation. The breeder's equation (*R* = *h^2^S*; Falconer & Mackay, 1996) provides a simplified illustration of this. Assuming swimming behaviour has a relatively low heritability (*h* ^2^ = 0.05; Visscher et al., 2008; Hansen et al., 2011), the estimate of winter–spring selection (*S* = β = −7.4) shifts the population's *p_10_* phenotype by *R* = −0.37. This is a greater change, in just one generation, than the difference in *p_10_* between *mean* and *deep* phenotypes (−0.18). Further research is needed to estimate the heritability of this behavioural trait, that is, to calculate the degree to which it is determined genetically, rather than by external factors. If heritability is high, then the industry must look towards evolutionarily informed strategies for managing lice across the farm network.

The average horizontal transport distance was slightly longer for the shallower phenotypes, as might be expected from increased wind‐driven dispersal near the surface (Johnsen et al., 2016). However, the differences were relatively small (1–2 km, or <9%) and the temporal averaged patterns of horizontal dispersal were very similar for phenotypes (Figure 3). Reductions in larval transmission distance or increases in the distance between farms can impact the lice connectivity of farms. However, these changes in distance generally need to be much larger (tens of kilometres) to significantly alter the connectivity network (Samsing et al., 2019).

### 4.3 Variation in infestation pressure

The infestation pressure (# infective copepodids grid^−1^ hour^−1^) across all depths in winter was only ~1% of that in spring (Figure 3). Because louse development is slower at colder temperatures, more lice died (under the 17% background mortality) or were flushed into the open ocean as nauplii in winter before they reached the copepodid stage. In addition, the modelled infectivity of copepodids is reduced under lower temperatures (Skern‐Mauritzen et al., 2020). The simulated number of copepodids residing in the water does not reflect actual louse infestation pressure over these periods, as the release of lice particles was kept constant in our simulations, whereas in reality it varies seasonally with the number of lice in the farms and the temperature‐dependant hatching rate. Indeed, the farm infestation pressure in Hardangerfjord in 2018 was as high in winter–spring as in spring–summer, evident from the number of new infestations (as chalimus salmon^−1^) reported on salmon farms at that time (www.barentswatch.no).

Louse particles were concentrated in the narrow confines of fjords and dispersed over greater areas at the coast and in the open ocean (Figure 3). This meant the infestation pressure at coastal farms was less than half of that at inner‐fjord farms (Figure 3). Although this was assuming all farms release identical quantities of larvae, simulations with more realistic releases of larvae have also found higher infestation pressures within fjords (Johnsen et al., 2016; Sandvik et al., 2020). In this context, the point of interest was the differences in infestation pressure between phenotypes, within each season and location. In winter–spring, lice of the *deep* and *shallow* phenotypes reached infestation pressures ~1.2 times and ~0.9 times that of the *mean* phenotype, respectively. This suggests that even in the absence of depth‐based preventions, there may be some directional selection for the *deep* phenotype during winter–spring. During this period, temperatures were warmer deeper in the water column, which shortens the development time to the copepodid stage and improves the attachment success of copepodids (Samsing, Oppedal, et al., 2016; Skern‐Mauritzen et al., 2020). Despite the advantages of occupying warmer water, copepodids in the laboratory do not adjust their depth in response to temperature, but rather aggregate at the surface, regardless of temperature (Crosbie et al., 2020). This suggests that over the copepodids' evolutionary history, the benefits of being near the surface, where wild salmonids typically swim (LaBar et al., 1978; Plantalech Manel‐La et al., 2009; Rikardsen et al., 2007; Strøm et al., 2018), outweighed benefits of temperature‐seeking behaviour. Today, however, many emerging depth‐based preventions shift farmed salmon into deeper water (Bui et al., 2020; Frenzl et al., 2014; Geitung et al., 2019; Glaropoulos et al., 2019; Korsøen et al., 2012). If these strategies become widespread, deep‐swimming copepodids in winter–spring would benefit from both improved temperatures and an increase in host availability.

### 4.4 Spatial and temporal variation in selection

Although barrier cages consistently selected for the *deep* phenotype, the strength of this selection varied temporally and spatially. Selection was stronger in spring–summer than in winter–spring, and stronger at coastal sites than at inner‐ and mid‐fjord locations (Figure 5). The differences in relative fitness were an effect of surface salinity. In our model, salinity avoidance overrode other behaviours and was identical for all phenotypes. This meant that where a thick brackish layer was present (most pronounced at inner‐fjord sites in spring), copepodids of all phenotypes were driven into deeper water, and the difference in fitness between phenotypes was reduced.

### 4.5 Applications

Our results can be applied under a framework of evolutionary principles to coordinate louse management strategies across farms in ways that protect their durability against resistance (Barzman et al., 2015; McEwan et al., 2016; Onstad et al., 2013; Zhan et al., 2015). Leaving large portions of the pest population unexposed to a selective pressure reduces the overall strength of selection on the population. These refugia can slow the rate of evolution or even halt it, if resistant phenotypes are lost (through genetic drift or fitness costs) as fast as they are selected for (Kreitzman et al., 2018). In the Pacific, large wild salmonid populations act as a refugia, providing a valuable ‘evosystem service’ to farms (Kreitzman et al. 2018). In the Atlantic, where farmed hosts greatly outnumber wild hosts (Dempster et al.), refugia can instead be farms that are left untreated through space or time (McEwan et al., 2015; REX Consortium, 2013). Deciding which farms do not receive the management strategy is a challenge, however, as there are multiple factors to consider. These include the connectivity of farms, the efficacy of the strategy and the strength of selection for resistant phenotypes.

Management strategies should be prioritized at farms that are highly connected in the network, that is, those receiving large quantities of lice from nearby farms and/or transmitting large quantities to new farms. Using effective prevention strategies at these sites essentially removes crucial nodes from the connectivity network and disrupts louse transmission through the area (Samsing et al., 2019). However, this relies on the strategy being effective. Our simulations predicted spatial and temporal variability in barrier efficacy. Using barriers at farms that are highly connected in winter–spring and on the coast is expected to have a greater effect on louse transmission than those in spring–summer and in fjords.

Our model results also highlighted a trade‐off between efficacy and selection pressure. Where barriers were more effective, they also imposed a stronger selection pressure, which may accelerate the evolution of resistance. Imposing strong selection at too many well‐connected farms also risks facilitating the spread of resistant genotypes through the population. Co‐ordinating management strategies is a complex process that must strike a balance between short‐term (minimizing infestation) and long‐term (extending the durability of the strategy) goals. To find an optimal approach, dispersal models need to be combined with epidemiological and evolutionary models (e.g. Groner et al., 2016; McEwan et al., 2016; Samsing et al., 2019).

### 4.6 Limitations

It is important to remember that we have not directly measured selection on farms, but have rather used a model to simulate the depth distribution and likely selection strength. A model‐based approach has logistical advantages but also some limitations, which highlight areas for future research and development. Firstly, lice particles were not modelled with phototactic behaviour, as in most previous version of the model (e.g. Johnsen et al., 2016; Sandvik et al., 2020). Coates et al. (2020) performed their behavioural assays in the dark, and so it is not known if phototaxis is influenced by family effects or pressure cues. Differences in depth between phenotypes might be amplified if there is also genetic variation in phototaxis, and selection could also act on this variation (Cousyn et al., 2001; Gliwicz, 1986; King & Miracle, 1995).

A second limitation is that our models assumed a background mortality rate of 17% day^−1^, taken from a study on a captive louse population (Stien et al., 2005). In nature, larval mortality at certain times of the year is likely to be much higher than this due to predation by planktivores (Brooker et al., 2018). Predation rates not only vary seasonally and geographically, but also with depth, potentially influencing the infestation pressure of copepodid phenotypes through space and time.

A third limitation is that our model did not capture the fine‐scale hydrodynamic processes that occur immediately around sea cages. For example, turbulence around cages with lice skirts can pull water down from 2.5 m and underneath 5 m skirts (Frank et al., 2015). Similarly, pressure differences can cause upwelling inside cages (Frank et al., 2015). These processes might affect the probability of different phenotypes passing underneath cage barriers. They might also influence how salmon semiochemicals are transported out of cages, which copepodids are attracted to over short distances (Bailey et al., 2006; Fields et al., 2017). Whether copepodids use host cues to navigate over longer distances (in a scale of metres) is unknown. More research is needed to determine what happens to larvae as they approach cages with depth‐based preventions.

Fourthly, limitations to salinity modelling might affect the relative importance of salinity gradients on selection. The NorKyst800 ocean model tends to have a sharper salinity gradient compared to observations (Asplin et al., 2020; Johnsen et al., 2016; Myksvoll et al., 2018) and so in reality, surface salinities might be lower and drive lice deeper than the model predicts. This may explain why our estimates of barrier efficacy (Figure 4) were slightly higher than those reported from experimental trials, when comparing similar areas and seasons (Geitung et al., 2019; Oppedal et al., 2017; Wright et al., 2017). By under‐estimating the effect of salinity on copepod depth, we may have over‐estimated the importance of behavioural phenotype, particularly at farms with a thick brackish surface layer. Other model simplifications included a fixed diffusion coefficient for vertical mixing (whereas mixing is in fact weaker with stronger stratification) and fixed larval salinity tolerance. There may be some genetic variation in salinity tolerance (Andrews & Horsberg, 2020), which would further complicate the vertical distribution of genotypes in the presence of salinity gradients.

### 4.7 Broader implications

A number of studies have used new data on salmon louse biology to update lice dispersal models, and compare their predictions with those from previous versions of the model (Crosbie et al., 2019, 2020; Johnsen et al., 2014; Skern‐Mauritzen et al., 2020). These have largely been concerned with more accurately predicting the transmission of lice through the environment. What has not been addressed is that larval traits included in these simulations might vary within the louse population. We have shown that dispersal models can also be used to address evolutionary questions. This can have potentially valuable applications for salmon aquaculture: insight into the strength of selection imposed by management strategies can be used to inform policy decisions aimed at mitigating the effects of louse evolution, to preserve the efficacy of these strategies. More broadly, physical barriers and other spatial manipulations have long been staples in terrestrial pest management (Boiteau & Verson, 2001), but using these strategies in an aquatic environment comes with unique challenges. Our findings suggest that preventative methods in aquaculture are not necessarily immune to adaptive ability demonstrated by pest species.

## CONFLICTS OF INTEREST

None declared.

## Supporting information

Fig S1‐S2

## Data Availability

Data for this study are available at: 10.5281/zenodo.4437577.
