# Supplementary material for: Parasite management in aquaculture exerts selection on salmon louse behaviour
Source: Evol Appl. 2021 Jun 2;14(8):2025–38. doi: 10.1111/eva.13255 (PMC8372093; doi:10.1111/eva.13255)

Supplementary Material

Supplementary Figure 1. Michaelis-Menton-type curves for the change in p_z (the probability of copepodids being in the 'swim' state) with depth, for 3 behavioural phenotypes (*mean*, *shallow* and *deep*); and their fit with data from experimental columns.

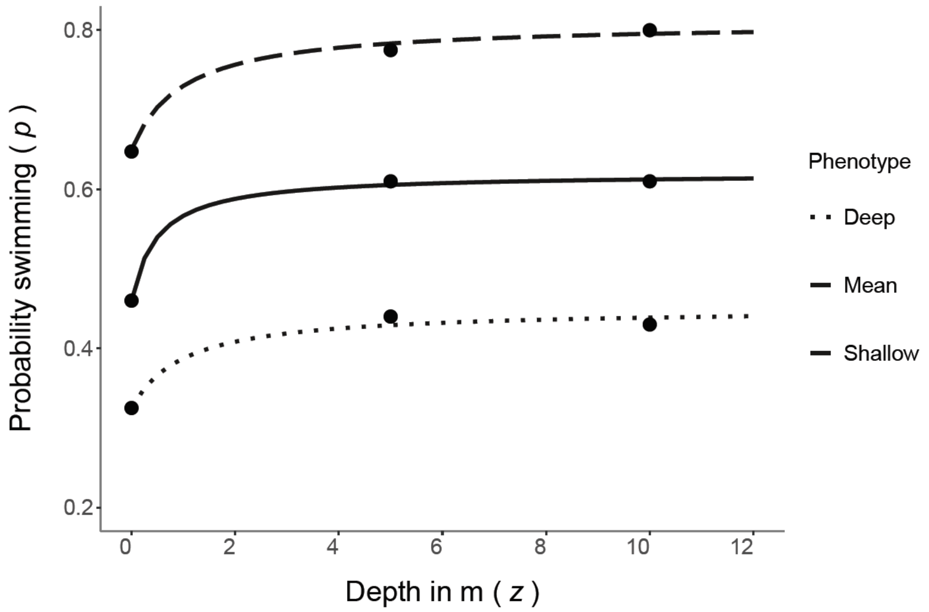

Supplementary Figure 2. Location of inner-fjord (red), mid-fjord (violet) and coastal (blue) areas (containing 10 farms each) in Hardangerfjord, Norway.

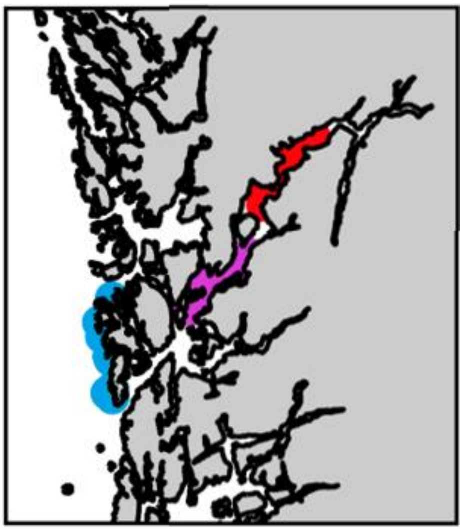

Supplement: Supplementary file 1 — Fig S1‐S2 [file EVA-14-2025-s001.pdf]
